# Usefulness of barium sulfate and iohexol as contrast agents for VFSS in visualizing components of swallowing predictable of poor outcomes

**DOI:** 10.1038/s41598-023-46297-4

**Published:** 2023-12-06

**Authors:** Min Soo Kang, Min Cheol Chang, Soyoung Kwak

**Affiliations:** 1 Department of Physical Medicine & Rehabilitation, College of Medicine, Yeungnam University, Daegu, South Korea; 2 Department of Physical Medicine & Rehabilitation, Yeungnam University Hospital, 170, Hyeonchung-ro, Nam-gu, Daegu, 42415 South Korea

**Keywords:** Outcomes research, Radiography

## Abstract

Barium sulfate and iohexol are commonly used as contrast agents for videofluoroscopic swallowing study (VFSS). This study compared their usefulness as contrast agents in visualizing components of swallowing predictable of subsequent pneumonia and unintentional weight loss after VFSS. This was a randomized, controlled, crossover trial. The two contrast agents were alternately used in the same participants, and the order in which the contrast agent was tested first was randomly assigned. After VFSS, we followed the participants for 3 months and the association between VFSS findings of each contrast agent and the subsequent pneumonia and unintentional weight loss were analyzed. A total of 30 participants were included in the analysis. We recorded 11 cases of subsequent pneumonia and 13 of unintentional weight loss. Regarding the risk of subsequent pneumonia after VFSS, only the oral transit time and number of swallows tested with barium sulfate indicated significant differences between participants with and without subsequent pneumonia. For unintentional weight loss, oral transit time and pharyngeal wall coating after swallowing tested with barium sulfate, as well as oral transit time, nasal penetration, residue in the valleculae, PAS scores, and number of swallows when testing with iohexol demonstrated significant differences between those with and without unintentional weight loss.

## Introduction

Dysphagia is difficulty or inability to form or move the alimentary bolus from the mouth to the stomach^1^. Specifically, oropharyngeal dysphagia refers to difficulties in oral preparatory, oral, and pharyngeal phases of swallowing, which include difficulty in biting, lip closure, chewing and mastication, mixing the food bolus with saliva, initiating swallowing, transporting the bolus to the esophagus, and removal of residue from the pharyngeal cavity^2^. There are two primary functional concerns regarding oropharyngeal dysphagia, namely the entry of food materials into the airway and the stasis of residue in the pharynx after a swallow^3,4^, and the consequences of oropharyngeal dysphagia can lead to pneumonia, weight loss, dehydration, and potentially death^2,4^. The prevalence of dysphagia in the adult population (aged > 18 years) was reported to be ≥ 16% and higher among the elderly^5,6^. Patients with dysphagia have higher prevalence rates of malnutrition, ranging from 45.3 to 88.2%, and malnutrition is significantly associated with a higher mortality rate of 42.4 to 70%^7,8^. Aspiration pneumonia, one of the most common causes of mortality, is significantly associated with dysphagia (up to 92%)^9,10^. Since dysphagia is strongly associated with mortality due to malnutrition and aspiration pneumonia, timely evaluation and appropriate treatment of dysphagia are crucial.

Videofluoroscopic swallowing study (VFSS) has been considered one of the gold standard assessments for evaluating dysphagia, as it can provide detailed information of the anatomical landmarks of the patient as well as swallowing physiology in real-time, which assists clinicians in determining the safety and efficiency of swallowing, developing a treatment plan, and providing appropriate dietary prescription for patients with dysphagia^11,12^.

Barium sulfate and iohexol are commonly used as contrast agents for VFSS^13–15^. Barium sulfate has low solubility in water and is not easily absorbed in the human body^16^. When a large amount of barium sulfate is aspirated, it can seriously compromise pulmonary function, which can be fatal, and even a small amount when aspirated can cause local inflammation, which might increase the incidence of pneumonia^17–20^. By contrast, iohexol is a water-soluble agent that can be easily absorbed in the human body and is known to have a relatively lower risk of pulmonary infection when aspirated^14,15,21^. These differences might result from the different characteristics of barium sulfate and iohexol, including their water solubility, viscosity, hardness, and adhesiveness. Considering that the physical characteristics of food can affect bolus transit time and velocity^22–26^, the different physical characteristics of barium sulfate and iohexol may also influence the detectability of abnormal findings during VFSS. Moreover, studies have reported that iohexol is not as dense as barium sulfate; thus, it might not detect all pharyngeal and esophageal pathology during VFSS^17^. However, most of the previous studies comparing the two contrast agents focused on the safety of the contrast agents during the VFSS^14,15,21^, and no study has ever compared the superiority between these contrast agents in assessing the severity of dysphagia and visualizing components of swallowing predictable of subsequent aspiration pneumonia and unintentional weight loss after VFSS.

The purpose of this study was to determine which contrast agent, barium sulfate or iohexol, is superior in visualizing the components of swallowing predictable of subsequent aspiration pneumonia and unintentional weight loss when used in the VFSS. The objectives of this study includes: (1) to compare VFSS findings when barium sulfate and iohexol was used as a contrast agent, (2) to compare whether there was a difference in the VFSS findings of participants with and without subsequent pneumonia by contrast agents used, (3) to compare the VFSS findings of participants with and without unintentional weight loss by contrast agents used, and (4) to compare the VFSS findings of participants who died and who survived by contrast agents used.

## Methods

This study was conducted in accordance with the Declaration of Helsinki and was reviewed and approved by the Institutional Review Board of the Yeungnam University Hospital (IRB NO.2021-07-021-002). Written informed consent was obtained from all participants. This study was not registered.

### Trial design

This study was a randomized controlled trial with a crossover design conducted from September 2021 to March 2022 in a tertiary university hospital. Because various characteristics of the patients are associated with the development of aspiration pneumonia and unintentional weight loss (e.g., age, need for bronchial suctioning, the presence of dysphagia and/or dehydration, muscle mass, catabolic conditions such as inflammation or infection, and underlying diseases of the patients)^22,23^, it is impractical to control for all the potential confounding factors. Instead, to minimize the effect of these confounding factors, we adopted a randomized controlled trial with crossover design.

**Figure 1:**
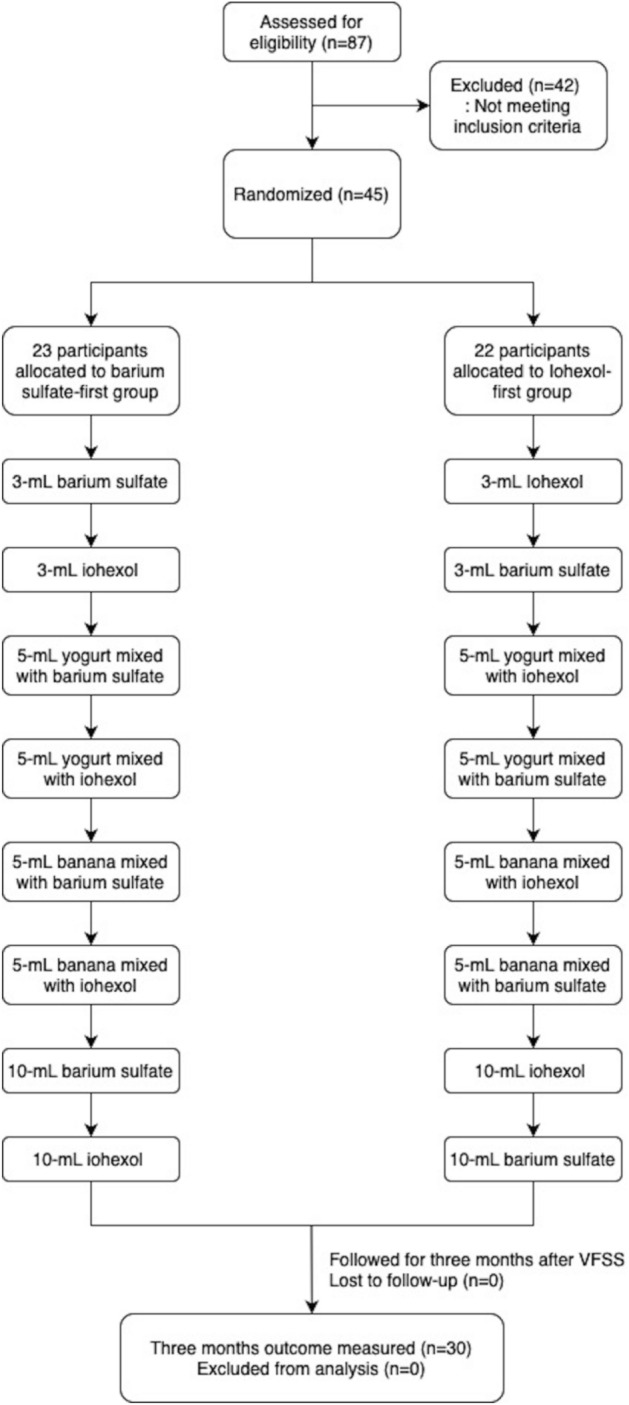
Design and flowchart of the study participants.

Iohexol (Bonorex 300, iohexol 647 mg/ml) and barium sulfate powder (Baritop HD, 99%w/w) 300 g mixed with 80 ml of water were compared in a singular VFSS. We postulated that the contrast agent that was previously used did not affect the subsequent VFSS findings, as it is assumed that previously tried food did not affect the later in the usual VFSS procedures, where various foods with different consistencies are tried in one examination^24^. During the procedures of the VFSS, the two contrast agents were alternately used during the VFSS without an additional washout period (Fig. 1). To minimize the carryover effect, after first VFSS using one contrast agent, second VFSS using the other contrast agent was conducted only when previously used contrast agent was not detected on fluoroscopic images. The block randomization method with block size of 4 and allocation ratio of 1:1 was performed for group assignment. One resident in rehabilitation medicine with 3 years of experience in VFSS and one radiographer with 20 years of experience performed the examination, and all the images obtained from the VFSS were analyzed frame by frame by two rehabilitation physicians with more than 10 years of experience in analyzing VFSS independently and no discrepancy between the two rehabilitation physicians was found. Blinding to the contrast agents used in the VFSS was not possible because the two contrast agents had different radiopacity and physical property, thus it was possible to determine which contrast agent was used by looking at the VFSS images.

Demographic data, including age, gender, and past medical history, were obtained from hospital records. In data collection, pneumonia was not included in pulmonary disease, and recent systematic infection history was defined as sepsis that occurred within 1 months of the date of VFSS. Initial dietary type was the type of diet at the time of VFSS. All participants were prospectively followed for 3 months after the VFSS. If the participant was still hospitalized at that point, data were obtained through hospital records, and if the participant was discharged, data was obtained by phone interviewing the participants or their guardian.

### Participants

The inclusion criteria were as follows: (1) patients who were admitted in a tertiary university hospital and referred to the Department of Physical Medicine and Rehabilitation for VFSS to evaluate the safety of swallowing, (2) patients who could maintain a sitting position during VFSS, (3) patients who could stay alert for at least 20 min, and (4) patients who have sufficient cognitive function to follow simple directions during VFSS. The exclusion criteria were as follows: (1) patients aged $<$ 20 years, (2) patients with laryngeal pathology including laryngeal cancer, stenosis, paralysis, presence or history of tracheostomy, and postoperative head and neck surgery, (3) patients who could not complete VFSS due to aspiration episodes more than once, and (4) patients who were newly diagnosed with other diseases that could aggravate dysphagia during the 3-month follow-up period.

All the inpatients who were referred to our department for VFSS were assessed for eligibility based on medical records. All participants were provided study-related information directly from the researchers and gave written informed consent 1 or 2 days prior to VFSS. Figure 1 presents the Consolidated Standards of the Reporting Trials (CONSORT) flow diagram.

### Intervention

VFSS was performed using an X-ray flat-panel detector system (FPD, Zexira®, Toshiba, Tokyo, Japan). Swallowing images were digitally recorded at a rate of 30 frames/s from a lateral viewing plane. The VFSS protocol consisted of (1) 3-ml thin liquid (barium sulfate solution or iohexol) provided with a spoon, (2) 5-ml yogurt mixed with each contrast agent provided with a spoon, (3) 5-ml banana mixed with each contrast agent provided with a spoon, and (4) 10-ml thin liquid (barium sulfate solution or iohexol) provided with a cup. Each of the consistencies was given twice to the participants, and the two contrast agents were given alternately for each time (a total of 8 boluses; 4 boluses mixed with barium sulfate and 4 boluses mixed with iohexol) (Fig. 1). Which contrast agent was used first was randomly assigned. To maintain a constant concentration of contrast agent to be mixed with yogurt and banana, 2 ml of each contrast agent was mixed with 10 g of yogurt and banana. According to IDDSI level, liquid barium sulfate solution was level 1, iohexol was level 0, yogurt mixed with each contrast agent were both level 4 and banana mixed with each contrast agent were both level 6. For patient safety, the VFSS was terminated if a second aspiration was observed at any stage of the VFSS. Imaging acquisition is maintained until the clearing of residue is completed. In addition, to minimize radiation exposure during VFSS, we performed VFSS at lateral viewing plane and total time for radiation exposure was controlled within 5 min by frequent ceasing of image acquisition^25^. If a radiation exposure time exceeded 5 min, VFSS was terminated and the participants were excluded from the analysis.

VFSS findings were categorized according to the penetration–aspiration scale (PAS)^26^ and functional dysphagia scale (FDS)^27^. The FDS is a 100-point scale originally developed for quantifying functional aspect of swallowing by supplementing PAS which measures only the anatomical depth of food material penetration^27^. Although it was initially developed for stroke patients, previous studies have revealed that it is also useful to evaluate dysphagia in patients with Parkinson’s disease, head and neck cancer, and swallowing disorders due to frailty or general weakness^6^. The highest score (higher scores indicate worse swallowing function) for any type of food tested on the VFSS was used in the analysis. Additionally, the efficiency of swallowing was evaluated using the number of swallows required to eliminate food from the pharyngeal space^28^. If the participants could not eliminate the contrast after repetitive swallows of over ten, the count was considered at ten. Since mild coating of pharyngeal space with contrast agent was considered as normal, only prominent residue in valleculae and pyriform sinuses were considered as remained bolus. We followed up the participants for 3 months after the VFSS and analyzed the association between the VFSS findings of each contrast agent and the risk of subsequent pneumonia and unintentional weight loss.

### Outcome measures

Development of subsequent pneumonia after VFSS during the follow-up period was the primary outcome of this study and secondary outcome was unintentional weight loss. A case of pneumonia was identified based on chest imaging and antibiotic prescription. Aspiration pneumonia is accepted as a part of the continuum of community-acquired pneumonia and hospital-acquired pneumonia rather than a distinct entity, and there have been no robust diagnostic criteria for the diagnosis of aspiration pneumonia^29^. In clinical settings, the diagnosis of aspiration pneumonia is generally made based on the combination of known risk factors for aspiration and involvement of characteristic location of the lungs in chest imaging^29^. In this study, all participants had known risk factors for aspiration (because they were referred for evaluating dysphagia), therefore, if the involvement of characteristic anatomical location of the lungs in the chest imaging with antibiotics prescription is confirmed, we regarded it as subsequent aspiration pneumonia. If the participant was discharged to another hospital and the chest imaging and antibiotic prescription could not be directly confirmed by researchers, it was checked by asking the participant or guardian if they had heard that from the medical staffs at the hospital they visited. Unintentional weight loss was defined as a loss of at least 5% of the usual body weight in $<$ 3 months^30^.

### Statistical analysis

This study is a preliminary study and required sample size was calculated using G*Power 3.1^31^. Assuming that the value of alpha to be 0.05, statistical power to be 95%, effect size to be 0.55, 39 participants were calculated to be needed. Due to the lack of preliminary studies on this topic, the effect size had to be chosen arbitrarily^32^. Assuming a dropout rate of 15%, a total of 45 participants were calculated to be needed.

Statistical analyses were performed using the Statistical Package for Social Sciences (version 22.0; IBM Corp., Armonk, NY, USA). The Wilcoxon signed-rank test was used to compare the VFSS findings (PAS, FDS, and number of swallows) for barium sulfate and iohexol. The Mann–Whitney test was used to compare the VFSS findings of each contrast agent in participants with or without subsequent pneumonia and unintentional weight loss. Receiver operating characteristic (ROC) curve analysis was performed to evaluate the predictive accuracy of VFSS findings for subsequent pneumonia and unintentional weight loss during the 3-month follow-up period. An area under the ROC curve (AUC) of 0.5, 0.7 to 0.8, 0.8 to 0.9, and more than 0.9 suggests no discrimination, acceptable diagnostic value, excellent diagnostic value, and an outstanding diagnostic value, respectively^33^. Statistical significance was set at* p* < 0.05.

## Results

A total of 45 participants were enrolled to this study, and 23 participants were enrolled to the group where barium sulfate was used as a contrast agent prior to iohexol, and 22 participants were enrolled to the group where iohexol was used prior to barium sulfate during the VFSS procedures (Fig. 1). Among them, 15 participants could not complete the VFSS due to aspiration episodes more than once, according to the VFSS protocol. Finally, 30 participants were included in the analysis (15 in barium sulfate-first group and 15 in iohexol-first group), and 11 cases of subsequent pneumonia (36.7%), 13 cases of unintentional weight loss (48.1%), and three cases of death (10%) were recorded during the follow-up period. All deaths were caused by sepsis due to pneumonia, and the cases of subsequent pneumonia included all the participants who died. The incidence of unintentional weight loss was analyzed including only 27 participants who survived. Among the 13 participants with unintentional weight loss, seven (54%) also experienced subsequent pneumonia. At 3 months after the VFSS, 13 participants were on a normal diet (48.2%), 10 participants were on a modified diet (37%), and 4 participants (14.8%) were on artificial feeding using a nasogastric tube. The demographic data of the participants and 3-month outcomes are presented in Table 1; no significant differences in the demographic characteristics and 3-month outcomes were observed between the groups tested for barium sulfate first and iohexol first (Table 1).

**Table 1:** Demographic data of the study participants.

Groups by first used contrast	Total	Barium sulfate first group	Iohexol first group	*p* value
Number of participants	30	15	15	
Age (years), mean ± SD	73.9 ± 9.7	77.3 ± 8.2	70.5 ± 10.2	0.081
≤ 69, n	9 (30%)	2	7	0.126
70–79, n	8 (26.7%)	5	3	0.367
80 ≥, n	13 (43.3%)	8	5	0.539
Gender, (men/women)	23/7	10/5	13/2	0.367
Stroke history, n	19 (63.3%)	7	12	0.126
Aspiration pneumonia history, n	17 (56.7%)	7	10	0.367
Other medical comorbidities				
Hypertension, n	16 (53.3%)	9	7	0.539
Diabetes mellitus, n	12 (40%)	5	7	0.539
Pulmonary disease, n	10 (33.3%)	8	2	0.061
Heart disease, n	5 (16.7%)	3	2	0.775
Degenerative brain disease, n	5 (16.7%)	4	1	0.367
Recent systemic infection history, n	10 (33.3%)	5	5	1.000
Initial dietary type				
Normal diet, n	4 (13.3%)	3	1	0.539
Modified diet, n	15 (50%)	9	6	0.367
Artificial feeding using nasogastric tube, n	11 (36.7%)	3	8	0.126
3-months outcome (n = 30)				
Pneumonia, n	11 (36.7%)	7	4	0.367
Death, n	3 (10%)	1	2	0.775
3-months outcome (n = 27)				
Participants still hospitalized, n	14 (51.9%)	7	7	1.000
Participants discharged home, n	13 (48.1%)	7	6	0.775
Unintentional weight loss, n	13 (48.1%)	7	6	0.867

When comparing the VFSS findings using barium sulfate and iohexol, no significant differences in PAS and FDS total scores were identified (Table 2). In the FDS subcategories, only “residue in pyriform sinuses” yielded higher scores when barium sulfate was used than when iohexol was used (*p* = 0.039). In addition, the number of swallows required to maximally eliminate food from the pharyngeal space was higher when barium sulfate was used than when iohexol was used (*p* = 0.005).

**Table 2:** Comparison of VFSS findings using barium sulfate and iohexol contrast agent.

	Barium sulfate, median (IQR)	Iohexol, median (IQR)	*p* value^a^
FDS score			
Total score	31 (11.5–51.0)	30 (10–46.3)	0.056
Lip closure	0 (0–0)	0 (0–0)	1.000
Bolus formation	0 (0–3)	0 (0–3)	1.000
Residue in oral cavity	0 (0–2)	0 (0–2)	1.000
Oral transit time	0 (0–6)	0 (0–6)	0.317
Triggering of pharyngeal swallow	10 (0–10)	10 (0–10)	1.000
Laryngeal elevation and normal epiglottic closure	0 (0–12)	0 (0–12)	0.655
Nasal penetration	4 (0–4)	4 (0–4)	0.180
Residue in valleculae	4 (4–8)	4 (0–8)	0.078
Residue in pyriform sinuses	4 (0–8)	4 (0–4)	0.039*
Coating of pharyngeal wall after swallow	0 (0–0)	0 (0–0)	0.180
Pharyngeal transit time	4 (0–4)	4 (0–4)	0.655
PAS score	3 (1–6)	3 (1–5)	0.162
Number of swallows to eliminate contrast from pharyngeal space	6 (3.75–10)	4 (2.75–6)	0.005*

*IQR* interquartile range, *FDS* functional dysphagia scale, *PAS* penetration aspiration scale.

^a^*p*-value was calculated by the Wilcoxon singed rank test. *p < 0.05.

Table 3 compares the VFSS findings between participants with and without subsequent pneumonia, tested using barium sulfate and iohexol as contrast agents. When analyzing PAS scores, FDS scores, and number of swallows required to maximally eliminate food from pharyngeal space, only the FDS subcategory of “oral transit time” (*p* = 0.048) and number of swallows (*p* = 0.005) when testing with barium sulfate had significantly higher values in participants with subsequent pneumonia than that in participants without subsequent pneumonia. When iohexol was used as a contrast agent, no significant differences in VFSS findings were observed between the participants with and without subsequent pneumonia.

**Table 3:** Comparison of VFSS findings between patients with and without subsequent pneumonia during 3-month follow up period.

Parameter (n = 30)	Barium sulfate			Iohexol		
	Patients with subsequent pneumonia (n = 11), median (IQR)	Patients without subsequent pneumonia (n = 19), median (IQR)	*p* value^a^	Patients with subsequent pneumonia (n = 11), median (IQR)	Patients without subsequent pneumonia (n = 19), medain (IQR)	*p* value^a^
FDS score						
Total score	50 (18–57)	22 (10–46)	0.077	44 (20–49)	26 (4–42)	0.106
Lip closure	0 (0–0)	0 (0–0)	0.609	0 (0–0)	0 (0–0)	0.609
Bolus formation	0 (0–3)	0 (0–3)	0.292	0 (0–3)	0 (0–3)	0.292
Residue in oral cavity	2 (0–2)	0 (0–2)	0.176	2 (0–2)	0 (0–2)	0.176
Oral transit time	6 (0–6)	0 (0–6)	0.048*	6 (0–6)	0 (0–6)	0.128
Triggering of pharyngeal swallow	10 (10–10)	10 (0–10)	0.192	10 (10–10)	10 (0–10)	0.192
Laryngeal elevation and normal epiglottic closure	12 (0–12)	0 (0–12)	0.354	12 (0–12)	0 (0–12)	0.163
Nasal penetration	4 (0–4)	4 (0–4)	0.759	4 (0–4)	0 (0–4)	0.269
Residue in valleculae	8 (4–8)	4 (4–8)	0.173	4 (4–8)	4 (0–4)	0.126
Residue in pyriform sinuses	8 (4–8)	4 (0–8)	0.053	4 (0–8)	4 (0–4)	0.172
Coating of pharyngeal wall after swallow	0 (0–10)	0 (0–0)	0.093	0 (0–0)	0 (0–0)	0.447
Pharyngeal transit time	4 (0–4)	4 (0–4)	0.287	4 (0–4)	4 (0–4)	0.424
PAS score	3 (1–7)	3 (1–6)	0.476	3 (2–7)	1 (1–3)	0.104
Number of swallows to eliminate food and contrast from pharyngeal space	10 (6–10)	5 (2–7)	0.005*	5 (4–6)	4 (2–6)	0.102

*IQR* interquartile range, *FDS* functional dysphagia scale, *PAS* penetration aspiration scale.

^a^*p*-value was calculated by the Mann–Whitney test. *p < 0.05.

Table 4 presents comparison of VFSS findings between participants with and without unintentional weight loss during the 3-month follow-up period. When barium sulfate was used as a contrast agent, the FDS subcategories of “oral transit time” (*p* = 0.013) and “coating of pharyngeal wall after swallowing” (*p* = 0.027) indicated significantly higher values in participants with unintentional weight loss, as well as the number of swallows (*p* = 0.017). Meanwhile, when iohexol was used as a contrast agent, the FDS subcategories of “oral transit time”, “nasal penetration”, and “residue in the valleculae” demonstrated significantly higher values in participants with unintentional weight loss than in participants without (*p* = 0.033, *p* = 0.027, and *p* = 0.036, respectively). Moreover, the PAS scores for cup drinking and number of swallows also demonstrated significantly higher values in participants with unintentional weight loss (*p* = 0.016 and *p* = 0.006, respectively).

**Table 4:** Comparison of VFSS findings between patients with and without unintentional weight loss during 3-month follow up period.

Parameter (n = 27)	Barium sulfate			Iohexol		
	Patients with unintentional weight loss (n = 13), median (IQR)	Patients without unintentional weight loss (n = 14), median (IQR)	*p* value^a^	Patients with unintentional weight loss (n = 13), median (IQR)	Patients without unintentional weight loss (n = 14), median (IQR)	*p* value^a^
FDS score						
Total score	45 (15–57.5)	23 (7–43.3)	0.152	41 (17–50)	24 (0–39.3)	0.080
Lip closure	0 (0–0)	0 (0–0)	0.937	0 (0–0)	0 (0–0)	0.937
Bolus formation	0 (0–3)	0 (0–3)	0.902	0 (0–3)	0 (0–3)	0.902
Residue in oral cavity	0 (0–2)	0 (0–2)	0.517	0 (0–2)	0 (0–2)	0.517
Oral transit time	6 (0–6)	0 (0–0)	0.013*	6 (0–6)	0 (0–0)	0.033*
Triggering of pharyngeal swallow	10 (5–10)	5 (0–10)	0.272	10 (5–10)	5 (0–10)	0.272
Laryngeal elevation and normal epiglottic closure	0 (0–12)	0 (0–12)	0.588	12 (0–12)	0 (0–12)	0.353
Nasal penetration	4 (2–4)	4 (0–4)	0.207	4 (2–4)	0 (0–4)	0.027*
Residue in valleculae	4 (2–8)	4 (4–8)	0.776	4 (4–8)	4 (0–4)	0.036*
Residue in pyriform sinuses	4 (2–8)	4 (0–8)	0.720	4 (2–6)	2 (0–4)	0.190
Coating of pharyngeal wall after swallow	0 (0–10)	0 (0–0)	0.027*	0 (0–0)	0 (0–0)	0.335
Pharyngeal transit time	4 (0–4)	4 (0–4)	0.820	4 (2–4)	2 (0–4)	0.155
PAS score	3 (1–6.5)	3 (1–6.3)	0.581	3 (2.5–7)	1 (1–3)	0.016*
Number of swallows to eliminate food and contrast from pharyngeal space	10 (5–10)	4.5 (2–7)	0.017*	5 (4–7)	3 (2–4.5)	0.006*

*IQR* interquartile range, *FDS* functional dysphagia scale, *PAS* penetration aspiration scale.

^a^*p*-value was calculated by the Mann–Whitney test. *p < 0.05.

To predict subsequent pneumonia, the AUC of the number of swallows when testing with barium sulfate was 0.806 (*p* = 0.006) (Table 5). When predicting unintentional weight loss, the AUC for “oral transit time’ and number of swallows when testing with barium sulfate were 0.736 and 0.766, respectively (*p* = 0.037 and *p* = 0.019) (Table 6). By contrast, when iohexol was used as a contrast agent in VFSS, the AUC of the PAS scores for cup drinking was 0.761 (*p* = 0.021), while that of the number of swallows was 0.805 (*p* = 0.007) (Table 6).

**Table 5:** Prediction of subsequent pneumonia, ROC analysis.

Parameter (n = 30)	AUC-ROC	*p* value	95% CI	
			Upper limit	Lower limit
Barium sulfate				
FDS score—oral transit time	0.687	0.093	0.891	0.482
Number of swallows to eliminate contrast from pharyngeal space	0.806**	0.006*	0.963	0.649

*AUC* area under the curve, *FDS* functional dysphagia scale, *PAS* penetration aspiration scale.

*p < 0.05, **AUC-ROC > 0.7.

**Table 6:** Prediction of unintentional weight loss, ROC analysis.

Parameter (n = 27)	AUC-ROC	*p* value	95% CI	
			Upper limit	Lower limit
Barium sulfate				
FDS score—oral transit time	0.736**	0.037*	0.933	0.540
FDS score—coating of pharyngeal wall after swallow	0.654	0.174	0.867	0.441
Number of swallows to eliminate contrast from pharyngeal space	0.766**	0.019*	0.950	0.583
Iohexol				
FDS score—oral transit time	0.698	0.081	0.903	0.493
FDS score—nasal penetration	0.720**	0.052	0.917	0.523
FDS score—residue in valleculae	0.720**	0.052	0.918	0.522
PAS score	0.761**	0.021*	0.951	0.571
Number of swallows to eliminate contrast from pharyngeal space	0.805**	0.007*	0.974	0.636

*AUC* area under the curve, *FDS* functional dysphagia scale, *PAS* penetration aspiration scale.

*p < 0.05, **AUC-ROC > 0.7.

## Discussion

No significant differences were observed in the PAS and FDS total scores according to the contrast agent used in the test. This might suggest that the difference in physical properties of the two contrast agents is not critical enough to make a difference in the anatomical depth to which food bolus penetrates when ingested. However, significant differences between the two contrast agents were seen in the FDS subcategories, scores for “residue in pyriform sinuses”. The reason for this difference between PAS and FDS scores could be because the FDS was developed to supplement PAS and it can report more qualitative aspects of VFSS findings than PAS. Additionally, a greater number of swallows were required with barium sulfate than with iohexol. According to a previous study, iohexol demonstrated a faster transit time in gastrointestinal studies than barium sulfate because iohexol has higher water solubility^34^. Furthermore, barium sulfate is reportedly more viscous than iohexol^35^. Therefore, higher scores of “residue in pyriform sinuses” in FDS and a greater number of swallows when using barium sulfate than when using iohexol might be due to iohexol having higher water solubility and lower viscosity than barium sulfate. However, considering the small effect size of the difference between scores for “residue in pyriform sinuses”, the result should be carefully interpreted.

Our study reveals that a longer oral transit time and greater number of swallows when testing with barium sulfate were associated with the development of subsequent pneumonia after VFSS. Of note, when these two factors were analyzed using ROC analysis, only the number of swallows demonstrated an excellent diagnostic value for predicting subsequent pneumonia. According to previous studies, prolonged swallowing time, including oral transit time, is associated with an increased risk of aspiration pneumonia^6,36^. Moreover, the amount of post-swallowing residue in the pharyngeal space after swallowing is known to be associated with a greater risk of airway penetration or aspiration^37^. Our findings are consistent with those of previous studies; however, in the ROC analysis, a significant differences was observed only in the number of swallows when barium sulfate was used. The reason why the number of swallows emerged as a stronger predictor than PAS scores or residue after swallowing, which are well-known predictors of pneumonia, may be the VFSS protocol implemented in this study. There is still no clear consensus on when to measure pharyngeal residue after swallowing, for example, after first swallowing response or after multiple swallowing response. We measured the residue after the participants tried multiple swallows in order to maximally eliminate residues from the pharyngeal space through multiple swallowing. Consequently, the amount of residue might be underestimated during the VFSS compared to a typical meal time in a hospital room or at home because it is difficult to visually check the amount of residue in the pharyngeal space and this might hinder the caregivers from encouraging multiple swallows. Instead, the number of swallows may better indicate the impaired ability to eliminate food bolus from the pharyngeal space. In addition, this significant result was found only when barium sulfate was used as a contrast agents; this might be due to the abovementioned different characteristics of the two contrast agents.

Our study also found significant differences in VFSS findings including oral transit time, coating of the pharyngeal wall after swallowing, and number of swallows in participants with and without unintentional weight loss when using barium sulfate. By contrast, when using iohexol, oral transit time, nasal penetration, residue in the valleculae, PAS scores, and number of swallows demonstrated significant differences between those with and without unintentional weight loss. Among these findings, oral transit time and the number of swallows when using barium sulfate had acceptable diagnostic values for predicting unintentional weight loss; however, the number of swallows when using iohexol may have an excellent diagnostic value for predicting unintentional weight loss, while the PAS scores when using iohexol had an acceptable diagnostic value. Similarly to subsequent pneumonia discussed earlier, due to the VFSS protocol of our study, oral transit time and number of swallows are thought to be able to better indicate the efficiency of swallowing compared to other metrics. Additionally, it is well known that unintentional weight loss is significantly associated with dysphagia^38^. Our findings are in accordance with those of a previous study in that seven out of 13 participants (54%) with unintentional weight loss also had subsequent pneumonia during the follow-up period. Moreover, the VFSS findings that revealed significant differences between participants with and without unintentional weight loss were consistent with those of previous reports^28,38,39^. Interestingly, the number of swallows was identified as a significant predictor of weight loss regardless of the use of either barium sulfate or iohexol as a contrast agent for VFSS, which has an acceptable diagnostic value for barium sulfate and an excellent diagnostic value for iohexol. This might be because the number of swallows reflects the global swallowing capacity and efficiency^28^. Another notable finding was that PAS scores were a significant predictor of unintentional weight loss only for iohexol. Considering that high-viscosity boluses have a reduced risk of aspiration during swallowing^40,41^, it might well be because barium sulfate is far more viscous than iohexol^35^.

To the best of our knowledge, this is the first study designed as a randomized, controlled, crossover trial to directly compare barium sulfate and iohexol as contrast agents for VFSS in predicting subsequent pneumonia and unintentional weight loss in patients with dysphagia. Because the two contrast agents were compared in the same participants, minimizing the influence of different patient characteristics on the results of the analysis was possible. Previous studies that compared barium sulfate and iohexol as contrast agents for VFSS focused on safety when the participants aspirated during the test, and since each contrast agent was administered to two different groups, the possibility of selection bias could not be completely excluded even if factors including age and sex were controlled.

However, this study has several limitations. First, the sample size was relatively small; future studies with larger sample sizes would provide more information for selecting the superior contrast agent between barium sulfate and iohexol. Second, underlying diseases of the participants were varied without restrictions. For example, although the characteristics and/or clinical course of dysphagia in patients with acute stroke and those with neurodegenerative diseases can be very different, our study was conducted without excluding specific diseases or targeting only specific diseases. To compare the ability of contrast agent to predict long-term prognosis of dysphagia, including various underlying diseases may have advantages in terms of generalizability, but the limitations due to this are also clear. Also, it should be noted that that although the statistical significance was not reached, more participants in barium sulfate-first group had pulmonary disease as medical comorbidity, which has close association with pneumonia and unintentional weight loss. Third, inability for blinding to the contrast agents used in the VFSS was another limitation of this study. Forth, the IDDSI levels of the two contrast agents were different, and this might well affect the study outcomes. Future study using equal IDDSI level of the liquid barium sulfate and iohexol would be necessary to better understand how the different physical properties of the two contrasts other than viscosity affect findings of VFSS. Fifth, subsequent pneumonia does not necessarily mean aspiration pneumonia. Despite efforts to include only aspiration pneumonia in the analysis, the possibility that other types of pneumonia may also be included cannot be completely ruled out. Sixth, although efforts were made to reduce the difference and make the conditions for testing the two contrast agents as similar as possible, muscle fatigue, which can be induced by performing multiple swallowing motions during the VFSS, may have had an effect for the results. To reduce the effect of fatigue, further study testing the two contrast agents in a differentiated participants would be needed. Last, we assumed that the previously administered contrast agent did not affect the subsequent VFSS findings; however, the lack of a washout period might have affected the study results. Although the previously used contrast agent was not detected using fluoroscopy, it might have affected the subsequent VFSS findings.

## Conclusion

In conclusion, barium sulfate could be superior to iohexol in predicting subsequent pneumonia, whereas iohexol could be superior to barium sulfate in predicting unintentional weight loss after VFSS. However, considering that iohexol could be safer than barium sulfate when aspirated, it might be a better option as a contrast agent for VFSS. In addition, understanding the influence of the physical properties of the contrast agent on VFSS findings can help clinicians in making better clinical decisions.

## Data Availability

The data that support the findings of this study are available from the corresponding author upon reasonable request.
